# Effects of 12 Weeks of Calanus Oil Supplementation on Cardiac Diastolic Function in Obese and Prediabetic Women—A Pilot Study

**DOI:** 10.3390/metabo15090596

**Published:** 2025-09-08

**Authors:** Felix Kerlikowsky, Fabian Spahiu, Eric J. Stöhr, Sina Junge, Wiebke Jonas, Edda van de Flierdt, Jan Philipp Schuchardt, Andreas Hahn

**Affiliations:** 1 Institute of Food Science and Human Nutrition, Leibniz University Hannover, 30167 Hannover, Germany; kerlikowsky@foh.uni-hannover.de (F.K.);; 2 COR-HELIX (CardiOvascular Regulation and Human Exercise Laboratory—Integration and Xploration), Institute of Sport Science, Leibniz University Hannover, 30167 Hannover, Germany

**Keywords:** Calanus oil, diastolic function, insulin sensitivity, metabolic health

## Abstract

**Background/Objectives**: In early-stage diabetes, diastolic dysfunction is an initial indicator of heart failure and is linked to altered glucose metabolism, including in prediabetes. Based on initial evidence that Calanus oil, derived from Calanus finmarchicus, which is rich in omega-3 polyunsaturated fatty acids and other bioactive compounds, benefits metabolic and cardiorespiratory health, this proof-of-principle study aimed to assess whether Calanus oil improves diastolic function in prediabetic women. **Methods**: Twenty middle-aged, obese women with prediabetes and no history of cardiac complications were enrolled and received 4 g/day of Calanus oil, providing 276 mg EPA + 256 mg DHA, for 12 weeks. Systolic and diastolic cardiac function, including the E/A ratio (E/A), was assessed by echocardiography. In addition, central blood pressure (BP) and pulse wave velocity (PWV) were analyzed by oscillometry. Metabolic health was evaluated using composite markers, including the metabolic syndrome severity score (Met-S score) and the triacylglycerol glucose–waist-to-height ratio (TyG-WHtR). **Results**: E/A was significantly improved (*p* = 0.023) following 12 weeks of Calanus oil supplementation. Furthermore, a significant improvement in metabolic health, indicated by a reduced Met-S score and a lower TyG-WHtR, was noticed (*p* < 0.001, respectively), reflecting decreased metabolic syndrome severity and enhanced insulin sensitivity. In addition, a significant reduction in diastolic BP, resting heart rate (*p* = 0.047), but not PWV or systolic BP (all *p* > 0.05) was observed. The improvement in E/A was associated with improved insulin sensitivity, as reflected by a decrease in the TyG-WHtR (*p* = 0.014). **Conclusions**: These exploratory findings suggest that Calanus oil supplementation in pre-diabetic women might improve central diastolic haemodynamics, accompanied by an overall improvement in metabolic health. However, the absence of a placebo control group limits definitive conclusions.

## 1. Introduction

As of 2024, approximately 589 million adults aged 20 to 79 worldwide are living with diabetes, representing about 10.5% of the global adult population. By 2045, projections from the International Diabetes Federation (IDF) indicate that one in eight adults, approximately 783 million people, will be living with diabetes—representing a 46% increase from current figures. Type 2 diabetes mellitus (T2DM) accounts for the majority of these cases [1].

The precursor of T2DM, known as prediabetes, is characterized by dysglycemia, in which the markers of glucose metabolism are elevated but still below the clinical threshold for a full T2DM diagnosis. Thus, prediabetes represents an elevated risk that has significant potential for the future development of T2DM and diabetes-related disorders such as diabetic cardiomyopathy [2]. Several risk factors contribute to prediabetes, including genetics, obesity, physical inactivity, and poor dietary choices [3]. Implementing lifestyle modifications, such as regular exercise, dietary improvements, better sleep, and weight management, can significantly lower these risks and, in some cases, even reverse prediabetes [4]. In diabetic cardiomyopathy, diastolic dysfunction presents as one of the earliest signs of impending heart failure. A deeper understanding of the metabolic dysregulation underlying dysglycemia and cardiomyopathy is crucial for developing effective prevention and management strategies.

Calanus oil is a sustainable marine source of long-chain omega-3 polyunsaturated fatty acids (n3 PUFAs), derived from the lipid-rich copepod Calanus finmarchicus, which is harvested from the North Atlantic [5]. Furthermore, Calanus oil contains antioxidants such as astaxanthin, plant sterols, and fatty alcohols. Compared to n3 PUFA sources derived from fish oil, Calanus oil differs fundamentally and contains n3 PUFA in a unique binding form. In Calanus oil, 80% of FAs are bound as wax esters, whereas in fish oil they are primarily bound to triacylglycerols (TAGs) and in krill oil to phospholipids [6,7].

Preliminary evidence suggests that Calanus oil supplementation might antagonize hypertension, fibrosis, and lower inflammatory response in obese mice [8]. In addition, a previous study by Štěpán et al. (2021) examined the effects of exercise training alone or in combination with Calanus oil in older women. Both interventions improved cardiorespiratory performance, but only exercise training combined with Calanus oil supplementation resulted in an increase in stroke volume [9].

In general, n3 PUFAs, in particular, docosahexaenoic acid (DHA, 22:6n3) and eicosapentaenoic acid (EPA, 20:5n3), have been shown to possess cardiometabolic-promoting effects [10]. Higher EPA and DHA intake and elevated blood levels have been associated with TAG lowering [11], anti-arrhythmic [12], and anti-inflammatory effects [13], as well as improved endothelial function [14]. Furthermore, EPA and DHA can promote vasodilation, thereby enhancing endothelial function and contributing to overall cardiovascular health [15].

Available evidence from RCTs indicates that EPA+DHA supplementation reduces systolic blood pressure, while doses of ≥2 g/day also lead to a reduction in diastolic blood pressure [15]. Furthermore, it is known that EPA+DHA supplementation has a positive effect on left ventricular systolic function in patients with heart failure [16]. However, it is still unclear how individuals with preserved ejection fraction can benefit from Calanus oil supplementation, which contains EPA+DHA and several other bioactive compounds that may offer additional health benefits in terms of metabolic health and diastolic dysfunction. Given the aforementioned effects of Calanus oil, it is possible that it may reduce diffuse cardiac fibrosis and improve the elastance of the heart, thereby minimizing diastolic dysfunction. Accordingly, the aim of this pilot study was to investigate the effects of Calanus oil supplementation on diastolic cardiac function in obese, prediabetic women. The study encompassed a 12-week intervention period, including a daily intake of 4 g of Calanus oil.

## 2. Materials and Methods

### 2.1. Study Design and Study Participants

The present study was conducted as a proof-of-principle intervention trial with one treatment group. This is a sub-study of a randomized, placebo-controlled parallel group intervention study, whose original aim was to examine the effects of 12 weeks of Calanus oil supplementation at varying doses, with or without lifestyle intervention, on parameters of glucose and lipid metabolism, focusing specifically on the HOMA index in obese participants. The study was conducted at the Institute of Food Science and Human Nutrition and at the COR-HELIX laboratory of the Institute of Sport Science, Leibniz University Hannover, Germany, according to the guidelines of the Declaration of Helsinki and principles of Good Clinical Practice. The study was registered in the German Clinical Register (DRKS00030256). All participants gave informed consent prior to enrolment. A comprehensive description of the study procedure can be found in Kerlikowsky et al. [17]. Briefly, interested participants were screened for eligibility using a digital screening questionnaire. After the verification of inclusion criteria [female, age between 30 and 75 years, abdominal obesity characterized by body mass index (BMI) ≥ 28 kg/m^2^, waist circumference (WC) ≥ 88 cm for women] and exclusion criteria (e.g., consumption of n3 FA supplements, ≥ 2 portion of fish per week, severe or chronic diseases, pregnancy), participants were invited to the study site for blood testing. After verifying additional blood test-based inclusion criteria—including HOMA index ≥ 2.5, glucose from 100 mg/dL to 126 mg/dL, or HbA1c from 5.6% to 6.5%—a total of 266 participants were enrolled in the study. This pilot sub-study was designed as an exploratory mechanistic investigation to assess the feasibility and preliminary cardiac effects of the intervention. Due to logistical and resource constraints, echocardiographic data were collected only from a subset of 23 women who consented to participate. Given that the main study already includes a placebo group, this sub-study focused exclusively on detailed echocardiographic assessments in the intervention group. As a result, no placebo group was included in this sub-study. For data collection, two examination days were scheduled at the beginning (t_0_) and at the end of the intervention (t_12_).

### 2.2. Composition of Supplement

Participants were advised to take 4 g/day (8 capsules) of Calanus oil (Zooca^®^Lipids, Tromsø, Norway), divided throughout the day and taken with at least 200 mL of water and a meal, for 12 weeks, to ensure adequate fat digestion and absorption. The composition of Calanus oil is shown in Table 1. To assess compliance, participants received a defined number of capsules, which were counted at the end of the intervention at the t_12_ examination. A subject was judged as compliant if at least 85% of the capsules were taken.

### 2.3. Anthropometry

Height was measured using a stadiometer (Seca GmbH & Co., KG, Hamburg, Germany). WC was measured between the lowest rib and the highest hip bone at the narrowest part of the midsection using a tape measure. Body weight was measured digitally (Seca GmbH & Co., KG, Hamburg, Germany) to the nearest 0.1 kg (lightly dressed, without shoes). The body composition markers relative to fat mass, phase angle, and visceral fat mass were analyzed using an 8-point bioelectrical impedance analyzer (BIA, mBCA525, Seca Company, Hamburg, Germany). Prior to this measurement, participants were instructed to urinate and remove all jewelry. Participants were then instructed to lie down on a stretcher and rest for about 5 min to ensure a balanced distribution of body fluids. Pulse wave velocity (PWV) and augmentation index (Alx) were determined using the boso ABI-system 100 PWV, BOSCH+SOHN Jungingen, Germany, 2019, according to the manufacturer’s recommendations. All measurements were taken by a trained nutritionist.

### 2.4. Medication and Physical Activity

The amount of regular physical activity during the intervention was recorded at t_0_ and t_12_ using the Freiburg Physical Activity Questionnaire described by Frey et al. [18]. Participants were instructed not to change their dietary habits (especially regarding the intake of n3 PUFA-rich foods) or physical activity during the intervention period. Participants were advised to report any changes in medication during the intervention phase.

### 2.5. Blood Markers, Blood Pressure, and Aggregated Scores

Blood samples were taken from the participants after an overnight fast of at least 12 h, between 6:00 am and 10:00 am, ideally at the same time on both examination days. Venipuncture was performed on an arm vein using EDTA tubes, serum tubes, and Gluco Exact tubes (Sarstedt AG & Co., KG, Nümbrecht, Germany). The samples were stored at 5 °C and transported on the same day to an accredited and certified laboratory (Laboratory Group Dr. Kramer and Colleagues). Glucose levels were assessed using a photometric method (Beckman Coulter GmbH, Krefeld, Germany). Insulin levels were measured using an electrochemiluminescence immunoassay (ECLIA) with the cobas 801e system (Roche Diagnostics GmbH, Mannheim, Germany), while HbA1c was determined using high-pressure liquid chromatography (HPLC) (Tosoh Bioscience, Griesheim, Germany). TAG, high-density lipoprotein cholesterol (HDL-C), and low-density lipoprotein cholesterol (LDL-C) were analyzed from serum tubes using a photometric method (Beckman Coulter GmbH, Germany). Plasma concentrations of CRP were determined using a human Magnetic Luminex Assay (Bio-Techne, Abingdon, Oxon, UK) and a Magpix Luminex instrument (Luminex Corp, Austin, TX, US) according to the manufacturer’s instructions. Blood pressure measurements were conducted at the Institute of Sport Science. Participants were strictly required not to perform any physical activity prior to the examination. Blood pressure and pulse wave velocity were measured oscillometrically with the Mobil-O-Graph (IEM, Aachen, Germany) after a 10 min seated rest. Brachial blood pressure was recorded in the seated position with the cuff at heart level. Three consecutive measurements were obtained at 2 min intervals, and the arithmetic mean of the three measurements was used for both blood pressure and PWV in all analyses. The browser-based American Metabolic Syndrome (Met-S) Severity Calculator (https://metscalc.org/metscalc, accessed on 12 August 2024) was used to calculate Met-S severity scores for each subject. The calculated Met-S severity score was first described by Gurka and De Boer et al. and takes into account the following cardiovascular disease risk parameters: systolic blood pressure, TAG, HDL-C, fasting glucose, as well as information on sex, age, race/ethnicity, and weight [19]. As a result, a single value based on WC was calculated for each woman. To evaluate insulin resistance (IR), the Homeostasis Model Assessment (HOMA) index was used, based on the following formula: HOMA index = fasting glucose [mg/dL] × Insulin [μU/mL]/405 (Matthews et al., 1985) [20] and the triacylglycerol glucose–waist-to-height ratio (TyG-WHtR) was calculated according to the following formula: (1) TyG = ln(TAG [mg/dL] × glucose [mg/dL]); (2) WHtR= WC [cm]/body height [cm]; (3) TyG − WHtR = TyG × WHtR [21].

### 2.6. Echocardiography

Diastolic function of the left ventricle (LV) was examined using a commercially available ultrasound system (Vivid E95, GE HealthCare, Trondheim, Norway) equipped with a 4Vc probe. Left ventricular end-diastolic volume (LVEDV), left ventricular end-systolic volume (LVESV), left ventricular stroke volume (LVSV), left ventricular ejection fraction (LVEF), and longitudinal strain (LS) were analyzed by 2-D B-mode analysis, as well as speckle tracking and automated function imaging (AFI) of the four-chamber view. If speckle tracking was not possible due to insufficient image quality, the autoEF function was used. The same method was applied for the two measurement time points. Importantly, frame rates were kept the same between visits, and all parameters were obtained by averaging three consecutive cardiac cycles. The transmitral E/A ratio (E/A), deceleration time (DT), and isovolumic relaxation time (IVRT) were obtained from a pulsed wave Doppler signal between the tips of the mitral valve leaflets. In addition, E/e’ was obtained by adding the pulsed wave tissue Doppler imaging (TDI) results from the septal mitral annulus. Diastolic dysfunction was classified according to the 2016 ASE/EACVI (American Society of Echocardiography/ European Association of Cardiovascular Imaging) guidelines for evaluation of LV diastolic function in participants with normal LVEF [22]. Diastolic dysfunction was categorized by evaluating two key indicators: average E/e’ (>14) and septal e’ velocity (<7 cm/s), which go beyond the set cut-off values. Diastolic function was categorized as normal if both variables fell within the normal range. If only one of them exceeded the cut-off value, diastolic function was deemed indeterminate. Conversely, if both variables exceeded the cut-off, the presence of diastolic dysfunction was assumed. All offline analysis was performed using the EchoPAC Software (Version 204, GE HealthCare, Trondheim, Norway).

### 2.7. Statistical Analyses

Continuous variables are shown as mean ± standard deviation (SD), while qualitative variables are presented either as absolute or relative frequencies, or only in relative figures. All analyses were performed per protocol using GraphPad Prism statistical software (GraphPad Prism 5, San Diego, CA, USA). The Shapiro–Wilk test assessed normality of the data. Additionally, quantile–quantile plots provided a visual inspection of the distribution. Differences in normally distributed variables were evaluated using the paired t-test. A *p*-value < 0.05 was considered statistically significant. Linear regression models were used to examine associations between echocardiographic parameters and markers of metabolic health. One blood pressure measurement with the Mobil-O-Graph was incomplete and excluded from statistical analysis. For the evaluation of heart volumes, only 12 participants were included in the final analysis due to insufficient image quality in this population.

## 3. Results

### 3.1. Baseline Characteristics

Of the 23 recruited participants, 20 completed the study (Table 2). The three dropouts were caused by absence from the final examination (t_12_). The mean age of the participants was 59 ± 10 years, and the mean BMI was 34 ± 4 kg/m^2^. The cohort comprised predominantly non-smokers (95%), while 20% reported no medication use. Use of antihypertensive medication was reported by 55% of the total cohort. No participant reported a change in the medication during the study period.

### 3.2. Body Composition, Blood Markers, Blood Pressure, Physical Activity

After 12 weeks of Calanus oil intake, a decrease in WC and absolute fat mass was observed (Table 3: *p* = 0.049 and *p* = 0.003, respectively). Vice versa, total body water (TBW) and extracellular water (ECW) increased (*p* = 0.001; *p* = 0.040). Post intervention, fasting glucose and TAG concentrations were lower (*p* = 0.010 and *p* = 0.001, respectively), with no change in other parameters of glucose and lipid metabolism. Central diastolic blood pressure (DBP) and heart rate (HR) decreased (*p* = 0.048 and *p* = 0.047, respectively). Overall, we observed a significant improvement in metabolic health status. This is summarized by a significant decrease in the Met-S score based on WC, reflecting lower severity of the metabolic syndrome, as well as a significant decrease in the TyG-WHtR, reflecting improvement in insulin sensitivity (both *p* < 0.001). However, no significant differences were observed in CRP, central systolic BP, Alx, or PWV. No changes in physical activity levels were observed (Table S1).

### 3.3. Cardiac Function

Data on cardiac function are presented in Table 4. At baseline examination (t_0_), no subject had diastolic dysfunction according to our dual factor criteria (E/e’ [>14] and/or septal e’ velocity [<7 cm/s]). After the intervention, no differences in LV volumes were noted. Similarly, no alterations were detected in DecT or IVRT, and e’ velocity or E/e’ throughout the intervention period. However, E/A was significantly improved and shifted from 0.97 to 1.03 (*p* = 0.023).

### 3.4. Relationships Between Cardiac Function and Other Variables

**Figure 1:**
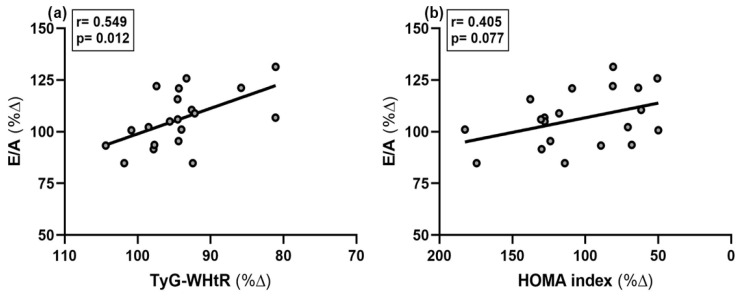
Association of the percentage change in E/A ratio (% Δ) and (**a**) percentage change in triacylglycerol glucose–waist-to-height ratio (TyG-WHtR) (% Δ) (**b**) percentage change in HOMA index (% Δ), n = 20.

At t_0_, E/A was significantly associated with TyG-WHtR, lower absolute fat mass, lower age, and lower PWV (all *p*-values < 0.05; ). The percentage increase in E/A was inversely associated with the percentage decrease in TyG-WHtR (Figure 1a), *p* = 0.012) but not with percentage changes in other metabolic health parameters (Table 5). However, a trend towards a decrease in the HOMA index and an increase in E/A was observed (Figure 1b), *p* = 0.077).

## 4. Discussion

This proof-of-principle trial demonstrated that 12 weeks of Calanus oil supplementation might lead to favorable changes in E/A, central DBP, and heart rate in prediabetic women. In addition, positive effects on parameters of metabolic health were observed.

The relationship between impaired glucose metabolism and left ventricular diastolic dysfunction is well established [23]. Hyperglycaemia promotes myocardial fibrosis and increased myocardial stiffness, leading to impaired diastolic relaxation [24]. Stahrenberg et al. (2010) found an elevated prevalence of subclinical diastolic dysfunction in prediabetic individuals [25]. Similarly, Di Pino et al. (2017) reported significantly lower E/A in prediabetic participants (1.10 ± 0.24) compared to healthy controls (1.18 ± 0.23) [26]. The participants in the present study started with an E/A of 0.97 ± 0.30, which is lower than that reported by Di Pino et al. for prediabetic participants and close to the lower limit of the normal range (0.8–2.0), suggesting a slightly impaired but not pathological diastolic relaxation pattern. The significant increase in E/A to 1.03 ± 0.30 after the intervention indicates an improvement in early diastolic filling. The improvement in E/A appears to be primarily driven by an increase in E-wave velocity, while A-wave velocity remained stable or slightly increased. This pattern typically reflects enhanced passive ventricular filling in early diastole, a possible marker of improved myocardial relaxation and compliance. These changes may, in part, be due to the observed reduction in central DBP (Table 3), which reduces ventricular afterload and facilitates early filling.

This is the first clinical trial that investigated the effects of Calanus oil supplementation on early-stage diabetic cardiomyopathy. However, preliminary evidence has shown that Calanus oil supplementation has beneficial effects on BP, inflammation, and fibrosis in obese mice []. Moreover, a number of animal-based studies have also revealed the beneficial effects of n3 PUFAs on diabetic cardiomyopathy in rats [27,28,29]. Mechanistically, EPA and DHA have been shown to support myocardial relaxation by reducing inflammation, improving endothelial function, inhibiting mitochondrial oxidative damage, increasing nitric oxide (NO) bioavailability, or through the autophagic pathway [30].

These effects might contribute to improved diastolic function in early stages of metabolic dysfunction, even in the absence of overt diastolic dysfunction. However, in this trial, CRP levels remained unchanged following Calanus oil supplementation, which may be attributable to the absence of elevated levels at baseline (< 3 mg/dL). Nevertheless, we observed a significant improvement in TyG-WHtR, a reliable surrogate indicator of IR [31]. This improvement was significantly associated with an improvement in the E/A, suggesting that it may reflect not only improved systemic glucose metabolism but also a shift in myocardial substrate utilization—specifically, from predominant fatty acid oxidation toward a more balanced or glucose-oriented energy metabolism. This warrants a brief explanation: in the healthy heart, energy is mainly produced via mitochondrial oxidative phosphorylation, with flexible substrate use. In insulin-resistant states, this flexibility is lost: fatty acid oxidation predominates, while glucose utilization declines due to impaired insulin signaling [32,33]. As fatty acid oxidation is less oxygen-efficient and promotes lipid intermediate accumulation (e.g., ceramides, diacylglycerols), it contributes to lipotoxicity, mitochondrial dysfunction, and altered calcium handling [34,35]. These changes are linked to impaired relaxation and increased myocardial stiffness, promoting diastolic dysfunction. In contrast, a more efficient energy supply through enhanced glucose metabolism could support active myocardial relaxation during early diastole, thereby improving ventricular compliance and E/A, even in the absence of overt clinical dysfunction. Additionally, we observed significant improvements in other parameters of metabolic health, including the aggregated marker Met-S score based on WC, heart rate, and central DBP.

The observed reductions in central DBP and heart rate further support the vascular and autonomic benefits of Calanus oil supplementation. Burhop et al. (2022) found no improvement in BP after 12 weeks of 2 g Calanus oil supplementation [36]. In this study, participants supplemented 4 g of Calanus oil, which may explain the stronger and more significant effects on central DBP. Several meta-analyses have already shown that n3 PUFA supplementation from fish oil is associated with improvements in BP [37,38]. A meta-analysis by Zhang et al. (2022) concluded that 2–3 g EPA+DHA per day is optimal to lower BP in healthy individuals, with stronger effects (>5 g/day) in those with dyslipidemia [39]. In the current study, the daily amount of supplemented EPA and DHA was significantly lower at 276 mg and 256 mg, respectively. Furthermore, there are also chemical differences between the n3 PUFA present in fish oil and Calanus oil. In Calanus oil, more than 80% of FAs are bound as wax esters, whereas in fish oil, they are primarily bound to TAGs. In addition, Calanus oil contains several other bioactive compounds, such as astaxanthin and policosanol. These compounds reduce oxidative stress and may, therefore, have beneficial effects on NO bioavailability, BP, and fibrosis [40].

### Limitations

This pilot study has some limitations that warrant recognition. A major limitation is the lack of a placebo-controlled design, which limits the ability to definitively attribute the observed effects to Calanus oil supplementation.

Statistical analyses are limited by the absence of multi-adjusted models that control for the possible influence of different ages, metabolic constellations, physical activity levels, and initial values of the examined parameters. However, given the small sample size, these models may lead to misinterpretation due to multicollinearity, overfitting, or the disproportionate influence of outliers, underscoring the need for further adequately powered placebo-controlled studies. Additionally, no formal correction for multiple testing was applied, increasing the risk of a type I error. However, this investigation was explicitly designed and labeled as an exploratory study to generate preliminary data on early-stage diabetic cardiomyopathy, resulting from Calanus oil supplementation. In exploratory studies, in which data are generated with an objective but not with a prespecified key hypothesis, multiple test adjustments are not strictly required and not recommended [41]. In contrast, multiple testing is more essential for confirmatory studies [41]. Moreover, this sub-study was underpowered for multiple formal comparisons. Finally, effect sizes and confidence intervals enable a robust interpretation of the results [42,43]. In addition, we did not assess the omega-3index (O3I) to evaluate the supply status of n3 PUFA at baseline and after 12 weeks of intervention. A significant association between an increase in EPA+DHA blood status and a change in E/A would contribute significantly to a better understanding of the variation in E/A and can also be used to monitor compliance. However, we screened all participants for low fish intake (less than 1x per week), which is a common approach to selecting participants with initial low O3I, as fish intake is the main dietary source of long-chain n3 PUFA. In addition, compliance was assessed by counting capsules at t_12_. This study lacks data on hormonal status. Altered levels of substrates and circulating hormones may potentially contribute to improvements in diastolic function. This aspect should be assessed in future studies.

Besides the E/A, multiple pre-specified outcomes related to metabolic and cardiac function were assessed. The image quality of the echocardiography data did not consistently allow a complete analysis. This issue arose due to the inherent physiological differences across individual participants. Therefore, the sample size was reduced for certain parameters of heart volume, collectively underscoring the need for cautious interpretation of the study’s outcomes and suggesting avenues for refinement in future investigations.

## 5. Conclusions

In summary, 12 weeks of Calanus oil supplementation showed potential to improve diastolic function by increasing E/A in prediabetic women. In addition, the supplementation of Calanus oil may have had a positive effect on metabolic health. While these exploratory results suggest metabolic and hemodynamic benefits, the underlying mechanisms and clinical relevance require further investigation, particularly due to the absence of a placebo group. Future studies should investigate the combined use of Calanus oil with lifestyle interventions in larger, more diverse, and placebo-controlled trials, including direct measures of myocardial metabolism by positron emission tomography.

## Figures and Tables

**Table 1:** Composition of Calanus oil.

Components	g/ 100 g Calanus Oil	mg/ 4 g Calanus Oil
MUFA	9.7	388
PUFA	26.2	1048
n3 PUFAs	25.0	1000
ALA	1.4	56
SDA	8.4	336
EPA	6.9	276
DHA	6.4	256
n6 PUFAs	1.1	44
LA	0.7	28
ARA	0.2	8
Fatty alcohols	28.8	1152
Sterols	0.35	14
Astaxanthin	0.1	4

Abbreviations: ALA, alpha-linolenic acid; ARA, arachidonic acid; DHA, docosahexaenoic acid; EPA, eicosapentaenoic acid; LA, linoleic acid; MUFA, monounsaturated fatty acids; PUFA, polyunsaturated fatty acids; SDA, stearidonic acid.

**Table 2:** Characterization of the study population (n = 20).

Parameters	Mean ± SD
**Age** [year]	59.25 ± 9.60
**BMI** [kg/m^2^]	34.24 ± 3.70
**Smoking status** Current smoker, n (%] Previous smoker, n %] Never smoke, n [%]	1 [5] 0 [0] 19 [95]
**Medical drug intake** No intake, n [%] Antihypertensive drug, n [%] other, n [%]	4 [20] 11 [55] 5 [25]

**Table 3:** Anthropometric, clinical variables, and laboratory parameters at t_0_ and at t_12_ examination.

	t_0_ (n = 20)	t_12_ (n = 20)	*p*-Value	Effect-Size, d (95%-CI)
**Variables**	**Mean ± SD**	**Mean ± SD**		
**Body weight** [kg] **WC** [cm]	94.52 ± 8.98 110.2 ± 9.66	94.18 ± 8.58 105.9 ± 10.47	0.242 0.049	0.022 (−0.43; 0.47) 0.573 (0.09; 1.04)
**Absolute fat mass** [%] **Visceral fat mass** [L] **TBW** [kg] **ECW** [kg]	44.37 ± 7.49 3.38 ± 1.21 38.01 ± 3.11 17.56 ± 1.38	42.75 ± 6.75 2.97 ± 1.21 38.86 ± 3.41 17.82 ± 1.45	0.003 0.023 0.001 0.040	0.874 (0.35; 1.33) 0.479 (0.01; 0.94) −0.578 (0.10; 1.05) −0.421 (0.01; 0.08)
**Fasting glucose** [mg/dL] **Fasting insulin** [µE/mL] **HOMA index** [AU] **HbA1c** [%]	106.3 ± 7.77 14.29 ± 6.58 3.77 ± 1.75 5.65 ± 0.30	103.7 ± 8.11 14.02 ± 5.89 3.60 ± 1.61 5.66 ± 0.28	0.010 0.418 0.310 0.360	0.572 (0.09; 1.04) 0.047 (−0.39; 0.49) 0.113 (−0.32; 0.55) 0.081 (−0.52; 0.36)
**TAG** [mg/dL]	151.9 ± 78.39	119.9 ± 45.37	0.001	0.782 (0.27; 1,28)
**TC** [mg/dL]	233.2 ± 47.24	236.45 ± 36.10	0.337	−0.294 (−0.75; 0.17)
**HDL-C** [mg/dL]	62.4 ± 11.74	63.7 ± 12.63	0.199	−0.328 (−0.79; 0.14)
**LDL-C** [mg/dL]	141.4 ± 33.04	139.9 ± 24.81	0.395	−0.080 (−0.53; 0.37)
**CRP** [mg/L]	2.78 ± 2.86	2.93 ± 3.52	0.358	−0.083 (−0.52; 0.36)
Central SBP * [mmHg]	130.8 ± 11.64	130.1 ± 13.33	0.372	0.842 (0.31; 1.36)
Central DBP * [mmHg]	81.08 ± 8.66	78.53 ± 9.22	0.048	0.814 (0.28; 1.32)
**HR** [bpm]	77.5 ± 8.71	74.43 ± 7.19	0.047	0.263 (0.72; 0.19)
**Alx** [%]	27.15 ± 10.69	29.15 ± 9.20	0.163	−0.195 (−0.65; 0.26)
**PWV** [m/s]	8.64 ± 1.35	8.6 ± 1.56	0.332	0.320 (−0.15; 0.78)
**Met-S-score WC** [AU]	0.76 ± 0.58	0.41 ± 0.53	0.001	1.344 (0.71; 1.96)
**TyG-WHtR** [AU]	6.34 ± 0.79	5.97 ± 0.76	0.001	0.930 (0.68; 1.50)

Abbreviations: AU: Arbitrary Unit; Alx, augmentation index; BMI, body mass index; CRP, c-reactive protein; SBP/DBP, systolic/diastolic blood pressure; ECW, extracellular water; HDL, high-density lipoprotein; HR, heart rate; LDL, low-density lipoprotein; Met-S-score WC: Metabolic syndrome severity score based on waistline; PWV, pulse wave velocity; TBW, total body water; TC, total cholesterol; TyG-WHtR, Triacylglycerol Glucose–Waist-to-Height Ratio. * n = 19. *p*-values in bold represent statistical significance at *p* < 0.05. d—Cohen’s d: standardized effect size expressing the mean difference between two time points. CI—Confidence Interval.

**Table 4:** Echocardiographic characteristics at t_0_ and at t_12_ examination.

Variables	t_0_ (n = 20)	t_12_ (n = 20)	*p*-Value	Effect-Size, d (95%-CI)
	Mean ± SD	Mean ± SD		
LV EDV * [mL]	100.72 ± 19.65	106.5 ± 17.32	0.166	−0.330 (−0.78; 0.18)
LV ESV * [mL]	46.03 ± 7.37	46.38 ± 9.03	0.908	−0.004 (−0.52; 0.44)
LV SV * [mL]	56.9 ± 13.56	60.72 ± 11.95	0.206	−0.280 (−0.76; 0.20)
LV EF * [%]	55.22 ± 5.39	56.31 ± 5.75	0.299	−0.21 (−0.68; 0.28)
LV CO * [L/min]	4.11 ± 0.96	4.12 ± 0.83	0.489	−0.010 (−0.47; 0.45)
**LS** [%]	−16.35 ± 2.88	−15.15 ± 2.98	0.116	−0.412 (−0.89; 0.07)
**DecT** [ms]	222.38 ± 30.29	218.28 ± 27.7	0.333	0.098 (−0.34; 0.54)
**E** [cm/s]	67.13 ± 13.72	69.67 ± 9.78	0.159	−0.212 (−0.069; 0.27)
**A** [cm/s]	71.28 ± 15.42	72.22 ± 16.51	0.338	−0.061 (−0.54; 0.42)
**E/A Ratio** [AU]	0.97 ± 0.30	1.03 ± 0.30	0.023	−0.479 (−0.94; −0.10)
**E’septal** [cm/s]	8.62 ± 2.04	8.82 ± 1.58	0.307	−0.046 (−0.48; 0.39)
**E/e’ septal** [AU]	8.00 ± 1.51	8.06 ± 1.38	0.419	−0.004 (−0.52; 0.44)
**IVRT septal** [ms]	81.12 ± 19.79	84.98 ± 21.38	0.240	−0.190 (−0.64; 0.29)
	**n (%)**	**n (%)**	***p*** **-value**	-
**Normal diastolic function**	18 (90)	18 (90)	0.500	
**Indeterminate diastolic function**	2 (10)	2 (10)	0.500	

Abbreviations: LV, left ventricular; EDV, end-diastolic volume; ESV, end-systolic volume; SV, stroke volume; EF, ejection fraction; LS, longitudinal strain; DecT, deceleration time; E, early ventricular filling velocity at the mitral valve; A, late ventricular filling velocity at the mitral valve; E/A, ratio of the early (E) to late (A) ventricular filling velocities; e’, early diastolic mitral annulus velocity; E/e’, early mitral inflow velocity to early diastolic mitral annulus velocity ratio; IVRT, Isovolumetric relaxation time; * n = 12. values in bold represent statistical significance at *p* < 0.05. d—Cohen’s d: standardized effect size expressing the mean difference between two time points. CI—Confidence Interval.

**Table 5:** Associations between the percentage change in E/A and the percentage change (% Δ) in markers of metabolic health.

% Δ Variables	r-Value	Beta-Coeff.	*p*-Value
% Δ TyG-WHtR % Δ HOMA index	0.549 0.405	−0.549 −0.405	0.012 0.077
% Δ Fasting glucose	0.189	−0.203	0.426
% Δ HbA1c	0.037	0.059	0.877
% Δ Met-S-score WC	0.127	−0.127	0.605
% Δ Central DBP *	0.080	0.080	0.665
% Δ Absolute fat mass	0.249	−0.249	0.290
% Δ Visceral fat mass % Δ Visceral fat mass	0.388 0.283	−0.235 −0.283	0.091 0.241

Abbreviations: TBW, total body water; TyG-WHtR, Triacylglycerol Glucose–Waist-to-Height Ratio; ECW, extracellular water; DBP, diastolic blood pressure; Met-S-score WC: Metabolic syndrome severity score based on waistline, * n = 19. Values in bold represent statistical significance at *p* < 0.05.

## Data Availability

Data are available upon request from the corresponding author.

## Supplementary Materials

The following supporting information can be downloaded at: https://www.mdpi.com/article/10.3390/metabo15090596/s1. Table S1: Questionnaire-based physical activity levels at t_0_ and at t_12_ examination.

## Abbreviations

The following abbreviations are used in this manuscript:

Alx	Augmentation index
AFI	Automated function imaging
BMI	Body mass index
DBP	Central diastolic blood pressure
BP	Central blood pressure
DT	Deceleration time
DHA	Docosahexaenoic acid
E/A	Ratio of the early (E) to late (A) ventricular filling velocities
EPA	Eicosapentaenoic acid
ECW	Extracellular water
HOMA	The Homeostasis Model Assessment
HR	Heart rate
HDL-C	High-density lipoprotein cholesterol
HPLC	High-pressure liquid chromatography
IR	Insulin resistance
IVRT	Isovolumic relaxation time
LDL-C	Low-density lipoprotein cholesterol
LV	Left ventricle
LVEF	Left ventricular ejection fraction
LVEDV	Left ventricular end-diastolic volume
LVESV	Left ventricular end-systolic volume
LVSV	Left ventricular stroke volume
LS	Longitudinal strain
Met-S score	Metabolic syndrome severity score
n3 PUFAs	Long-chain omega-3 polyunsaturated fatty acids
NO	Nitric oxide
O3I	Omega-3 index
PWV	Pulse wave velocity
