# Supplementary material for: Effects of 12 Weeks of Calanus Oil Supplementation on Cardiac Diastolic Function in Obese and Prediabetic Women—A Pilot Study
Source: Metabolites. 2025 Sep 8;15(9):596. doi: 10.3390/metabo15090596 (PMC12472083; doi:10.3390/metabo15090596)
Supplement: Supplementary file 1 [file metabolites-15-00596-s001.zip › metabolites-3793793-supplementary.pdf]

Table S1. Questionnaire based physical activity levels at t_0 and at t_{12} examination

	**t_0 (n = 18*)**	**t_{12} (n= 18*)**	**p-value**
**Variables**	**mean ± SD**	**mean ± SD**	
Basic activity [h/week]	4.05 ± 4.06	5.97 ± 4.44	0.089
Leisure activity [h/week]	2.51 ± 3.75	2.72 ± 4.08	0.276
Sports activity [h/week]	1.02 ± 1.83	1.78 ± 2.23	0.092

* Reduced data for statistical analysis due to incomplete questionnaires.
